# Comparative Evaluation of Marginal Adaptation and Micropermeability of Composite Resins With Different Fillers: An In Vitro Study

**DOI:** 10.7759/cureus.87694

**Published:** 2025-07-10

**Authors:** Dhivya Chandran, Anitha Thangaraju, Sankar Vishwanath, Sebeena Mathew, Karthick Kumaravadivel, Hema Kumari Thangavel

**Affiliations:** 1 Conservative Dentistry and Endodontics, KSR Institute of Dental Science and Research, Tiruchencode, IND; 2 Conservative Dentistry and Endodontics, KSR Institute of Dental Science and Research, TIRUCHENGODE, IND; 3 Conservative Dentistry and Endodontics, KSR Institute of Dental Science and Research, Tiruchengode, IND

**Keywords:** dental composite, dye penetration, marginal adaptation, microleakage, scanning electron microscope

## Abstract

Objective

To evaluate the marginal integrity of proximal cavities restored with three different types of composite, varying in fillers.

Methodology

Freshly extracted 84 intact human maxillary and mandibular premolars were used in the study, proximal cavities were prepared with a buccolingual dimension of 4 mm and mesiodistal dimension of 2 mm and the gingival seat was placed 1 mm below the cementoenamel junction (CEJ) and randomly divided into three groups: Group I (*n* = 28), NeoSpectra; Group II (*n* = 28), Tetric EvoCeram; and Group III (*n *= 28), Filtek Z250. They were restored with composites of experimental groups. The samples were subjected to thermocycling and dye penetration to evaluate marginal adaptation using scanning electron microscopy (SEM) and microleakage using a stereomicroscope.

Results

It was observed that an MQ4 score was present only in Group III, while an MQ1 score was more frequently observed in Group I. For microleakage, the expression of score 3 was higher in Group III with Tetric EvoCeram. NeoSpectra specimens predominantly showed scores of 1 and 2. There was no significant difference among the groups.

Conclusions

Within the limitations of this study, it is established that NeoSpectra outperformed other composites in marginal adaptation due to its pre-polymerized filler content. In terms of microleakage, there is not much difference between the groups, which may be attributed to their lack of active bond sites.

## Introduction

Dental composites are considered a paragon in restorative dentistry. Their supreme resistance and retention features allow for a minimal preparation design that conserves the maximum possible tooth structure. Resin-based dental composites exemplify aesthetic tooth-mimicking materials that form a relatively stable bond with tooth structure and yield acceptable clinical performance, rendering them indispensable in restorative dentistry [1].

Demarco et al., in a comprehensive review regarding the factors influencing the longevity of direct composite restorations, proposed that the survival rates of these composites vary from 23% to 97.7%. This disparity in the longevity of composites may be attributed to their susceptibility to technique sensitivity, which is closely linked to their handling properties, polymerization shrinkage, and limitations in achieving an adequate depth of cure, as well as the need for proper adhesive bonding with the tooth structure under diligent isolation [2].

The polymerization reaction causes volumetric shrinkage and internal stress to be created within the material. When this stress is close to or exceeds the local adhesive force, it forms gaps in the bonded interface, ultimately resulting in a mediocre seal at the interface of the tooth structure and the restoration [3].

Contraction stresses in the material can be overcome by altering the material formulation, such as monomeric chemistry and structure, fillers, interactions between the matrix and filler, and additional additives that employ different material polymerization strategies [4].

Filler particles form the inorganic portion of dental composites, and various modifications have been made to their particle size, dispersion pattern, configuration, microstructure, material composition, interfacial behavior, and porosity [5].

Owing to the inevitable role attributed to the filler segment in improving the mechanical properties of the composite [6]. One area of focus has been the refinement of filler technology. Nanotechnology, in particular, has revolutionized the field by allowing for the creation of nanofilled and nanohybrid composites with significantly smaller filler particles [7].

A composite with innovative filler technology, known as NeoSpectra, was introduced to address the issue of polymerization shrinkage while maintaining its favorable mechanical properties. Those pre-polymerized fillers exhibit low elastic modulus, allowing for greater flexibility within the resin matrix. This flexibility reduces internal stresses during polymerization, mitigating the risk of gap formation and subsequent microleakage. 

An increase in the filler concentration increases its pervasion into the resin, forming a dense mesh that amplifies the mechanical properties as long as there is no separation of the integrated filler-matrix system [6]. Hence, this in vitro study aimed to assess whether NeoSpectra could outperform two other nano-hybrids that are being used in clinical practice at present with respect to marginal adaptation and microleakage.

## Materials and methods

Table 1 represents the composition of materials used in the present study.

**Table 1:** Composition of materials used in this study.

Materials	Brand	Composition
Filtek Z250 xt	3 M ESPE, St. Paul, MN	Zirconia/silica without silane treatment; bisphenol A-glycidyl methacrylate (Bis-GMA), urethane dimethacrylate (UDMA), bisphenol A ethoxylated dimethacrylate (bis-EMA), and triethylene glycol dimethacrylate (TEGDMA).
Tetric EvoCeram bulk fill	Ivoclar Vivadent, Schaan, Liechtenstein	Bis-GMA, UDMA, bis-EMA, barium alumina silicate glass filler, ytterbium fluoride, spherical mixed oxide
NeoSpectra ST HV	Dentsply, Konstans, Germany	Methacrylate-modified polysiloxane (organically modified ceramic) dimethacrylate resins, ethyl-4-(dimethylamino) benzoate, and bis(4-methylphenyl)iodonium hexafluorophosphate. Filler load: 78%-80% by weight, consisting of spherical, prepolymerized SphereTEC fillers (d₃,₅₀ ≈ 15 μm), non-agglomerated barium glass, and ytterbium fluoride.

Specimen preparation

Ethical approval was obtained before commencement of the study. Eighty-four freshly extracted intact human premolars from both the maxillary and mandibular arches were included in the study. The specimens that were extracted for orthodontic and periodontal purposes were collected and stored in distilled water containing thymol crystals until use. Teeth with cracks, fractures, and anatomical abnormalities were excluded. The teeth were thoroughly rinsed with running water before cavity preparation for two minutes. The proximal cavities were prepared with a standardized dimension of buccolingual width 4 mm and mesiodistal width 2 mm. The gingival wall was placed 1 mm apical to the cementoenamel junction (CEJ), with straight fissure diamond abrasive burs (SF 31; Mani Inc., Tochigi, Japan). The dimensions of all the cavities were verified using a William graduated periodontal probe. Burs were operated at a high speed in an air rotor with water coolant. Burs were replaced after every fifth preparation to maintain their optimal performance. 

The prepared samples were randomly divided into three groups (*n *= 28 per group) and subjected to restoration with three different composite materials as follows:

Group I: NeoSpectra ST HV (Nano-hybrid composite with pre-polymerized resin fillers) Group II: Filtek Z250 XT (Nano-hybrid composite) Group III: Tetric EvoCeram (Nanofilled composite)

Restorative procedure

Acid etching of the cavity was performed with 37% phosphoric acid (Eco-Etch, Ivoclar Vivadent, Mumbai, India) for 20 seconds on both enamel and dentin. A total-etch dental adhesive (Tetric N-Bond, Ivoclar Vivadent, Mumbai, India) was applied, gently air-dried, and light cured using a polywave LED polymerization unit (Bluephase N, Ivoclar Vivadent, Schaan, Liechtenstein) with a power intensity of 1,200 mW/cm². Based on the groups allocated, restorations were done with the aforementioned composites, respectively. The packable composite material was placed and condensed into the prepared cavity using a composite filling instrument. Each increment, approximately 2 mm thick, was cured for 20 seconds. After completing the restoration, sequential finishing was performed using Shofu finishing and polishing disks (Shofu Dental Pvt. Ltd., New Delhi, India). The samples were then stored in distilled water for 24 hours before thermocycling.

Thermocycling

The preserved samples underwent thermocycling for 1,000 cycles, involving alternate immersion in water baths maintained at 5 ± 8 °C and 55 ± 8 °C. Each sample remained in each bath for 2 minutes, with a transfer time of 5 seconds between baths. After thermocycling, the teeth were thoroughly dried. Half of the specimens from each group were analyzed using scanning electron microscopy (SEM) to evaluate marginal adaptation, while the remaining half were assessed for microleakage using a stereomicroscope.

Dye penetration

The root ends were then sealed with sticky wax, and two coats of nail varnish were applied to envelop the entire tooth surface, leaving a precise 1 mm margin around the restoration. Subsequently, the teeth were immersed in a 2% buffered methylene blue dye solution for a duration of 24 hours under carefully controlled vacuum conditions to check for microleakage.

SEM investigation

For SEM analysis, the prepared samples were gold-coated using a low-vacuum cathodic sputtering device (JEOL JFC-1200, Peabody, MA) and examined under an SEM (JEOL JSM-5510, Tokyo, Japan) to directly evaluate the marginal seal of the restorations. Following a 24-hour immersion in a 2% methylene blue dye solution, the samples were meticulously examined at a magnification of 250x. Post-examination, the tooth samples were subjected to a comprehensive scoring and categorization process following the rigorous Blunck and Zaslansky scoring criteria (Table 2) [8].

**Table 2:** Scoring criteria for marginal adaptation.

Score	Scoring criteria
MQ1	Margin not or hardly visible; no or slight marginal irregularities; no gap
MQ2	No gap, but severe marginal irregularities
MQ3	Gap visible (hairline crack up to 2 µm); no marginal irregularities
MQ4	Severe gap (>2 µm); slight and severe marginal irregularities

Stereomicroscopic evaluation

After 24 hours of dye penetration, the remaining half of the samples were rinsed with running water, and the coating of nail varnish was cleared from the surface using a Bard-Parker (BP) blade. Longitudinal sections of the teeth, in the mesiodistal direction along the root's length, were obtained using a diamond disc. These tooth sections were then examined at the gingival margins near the tooth-restoration interface using a stereomicroscope set at 40x magnification. Scoring was performed based on the depth of dye penetration to evaluate microleakage. None of the teeth were fractured during sectioning, and all the restored specimens were found suitable for scoring.

Scoring criteria for microleakage evaluation

Scoring criteria for microleakage evaluation are as follows [9]:

Score 0: No dye penetration Score 1: Dye penetration less than one-third of the cavity depth Score 2: Dye penetration less than two-thirds of the cavity depth Score 3: Dye penetration into the entire cavity depth 

All the restorative procedures were performed by a single trained operator to avoid errors. Scoring for both parameters was performed by two independent trained examiners who were blinded to the test groups. Cohen’s κ test was performed to assess intra-examiner reproducibility.

## Results

**Figure 1:**
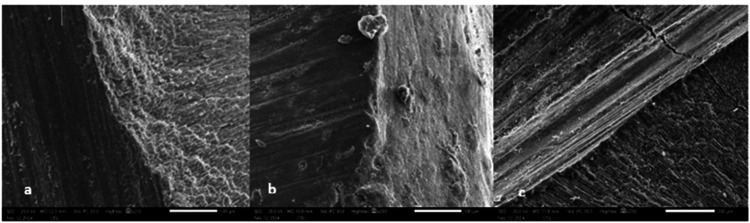
Scanning electron microscopy (SEM) images showing marginal adaptation of all three groups. (a) Marginal adaptation of NeoSpectra (b) Marginal adaptation of Filtek Z250 (c) Marginal adaptation of Tetric EvoCeram bulk fill

Figure 1 shows SEM images taken at a magnification of 250x.

Table 3 represents the intergroup comparison of scores for marginal adaptation, evaluated using the chi-square test. There is a statistical significance (*P* = 0.021*) for all groups comparing the mean percentage of scores obtained. It was observed that an MQ4 score was seen only in Group 3 (3, 21.43%), while an MQ1 score, indicating better adaptation, was more frequently observed in Group 1 (9, 64.29%).

**Table 3:** Comparison for marginal adaptation using the chi-square test. MQ1, score 1; MQ2, score 2; MQ3, score 3; MQ4, score 4. Group I, NeoSpectra; Group II, Filtek Z250; Group III, Tetric EvoCeram bulk fill. *Statistical significance (*P* < 0.05). *n*, number of samples with respective scores; %, percentage of samples with respective scores

Scores	Group I		Group II		Group III		Statistics	*P*-value
	n	%	n	%	*n*	%		
MQ1	9	64.29	4	28.57	1	7.14	6.59	0.021*
MQ2	4	28.57	6	42.86	4	28.57		
MQ3	1	7.14	4	28.57	6	42.86		
MQ4	0	0	0	0	3	21.43		
Total	14	100	14	100	14	100		

**Figure 2:**
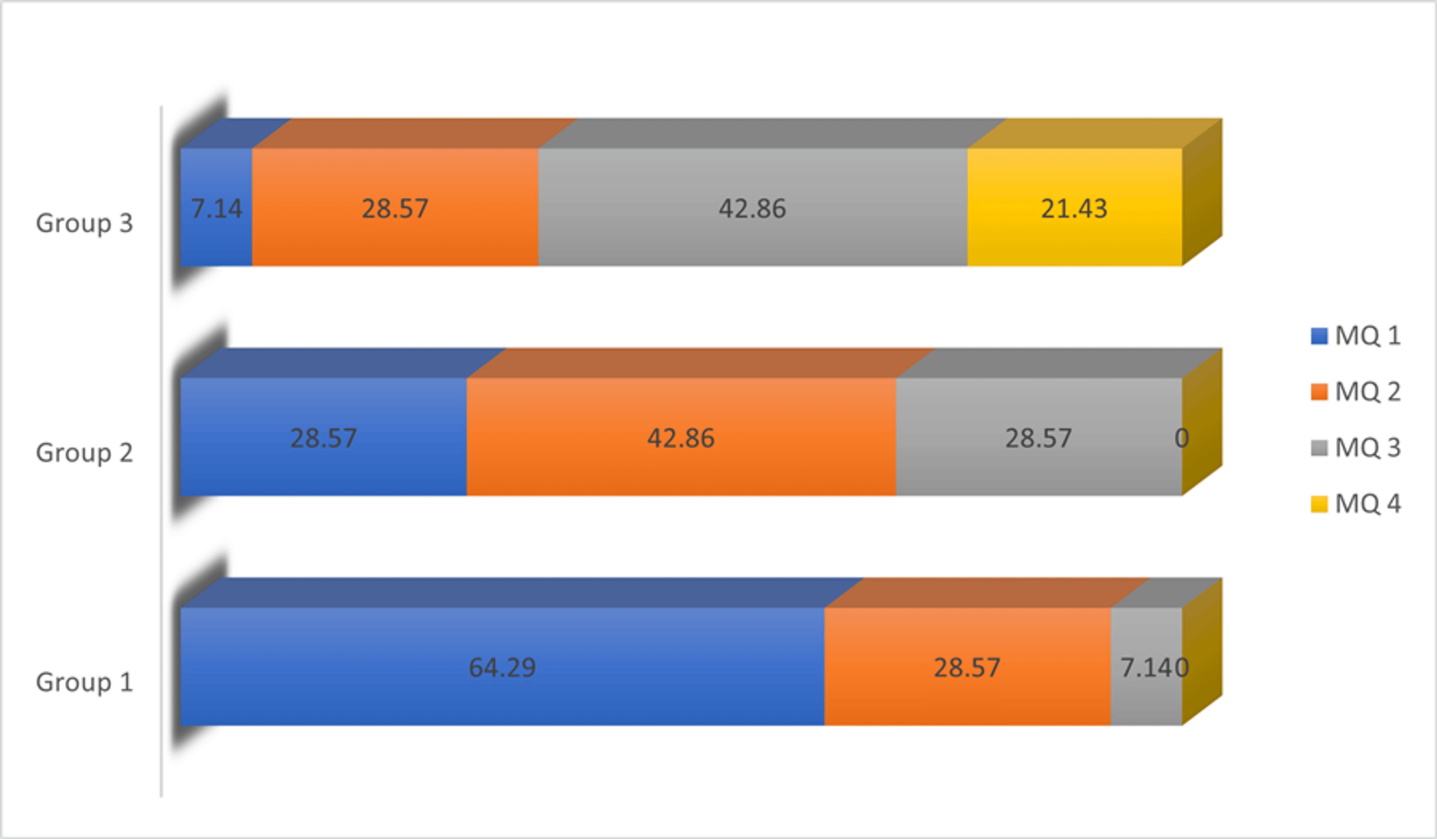
Graphical representation of marginal adaptation scores in percentage. Group I, NeoSpectra; Group II, Filtek Z250; Group III, Tetric EvoCeram bulk fill. MQ1, score 1; MQ2, score 2; MQ3, score 3; MQ4, score 4.

Figure 2 depicts a graph representing the score distribution across each group.

Table 4 indicates an intergroup comparison of scores of dye penetration evaluated using the chi-square test. There was no significant difference among the groups.

**Table 4:** Intergroup comparison of microleakage scores analyzed using the chi-square test. Group I, NeoSpectra; Group II, Filtek Z250; Group III, Tetric EvoCeram bulk fill. *n*, number of samples with respective scores; %, percentage of samples with respective scores Score 0, no dye penetration; score 1, dye penetration less than one-third of the cavity depth; score 2, dye penetration less than two-thirds of the cavity depth; score 3, dye penetration into the entire cavity depth.

Score	Group I		Group II		Group III		Statistics	*P*-value
	n	%	n	%	n	%		
No dye penetration	4	28.6	2	14.3	0	0	10.466	0.086
<1/3rd of cavity depth	5	35.7	3	21.4	2	14.3		
<2/3rd of cavity depth	5	35.7	6	42.9	7	50		
Dye penetration entire cavity depth	0	0	3	21.4	5	35.7		

**Figure 3:**
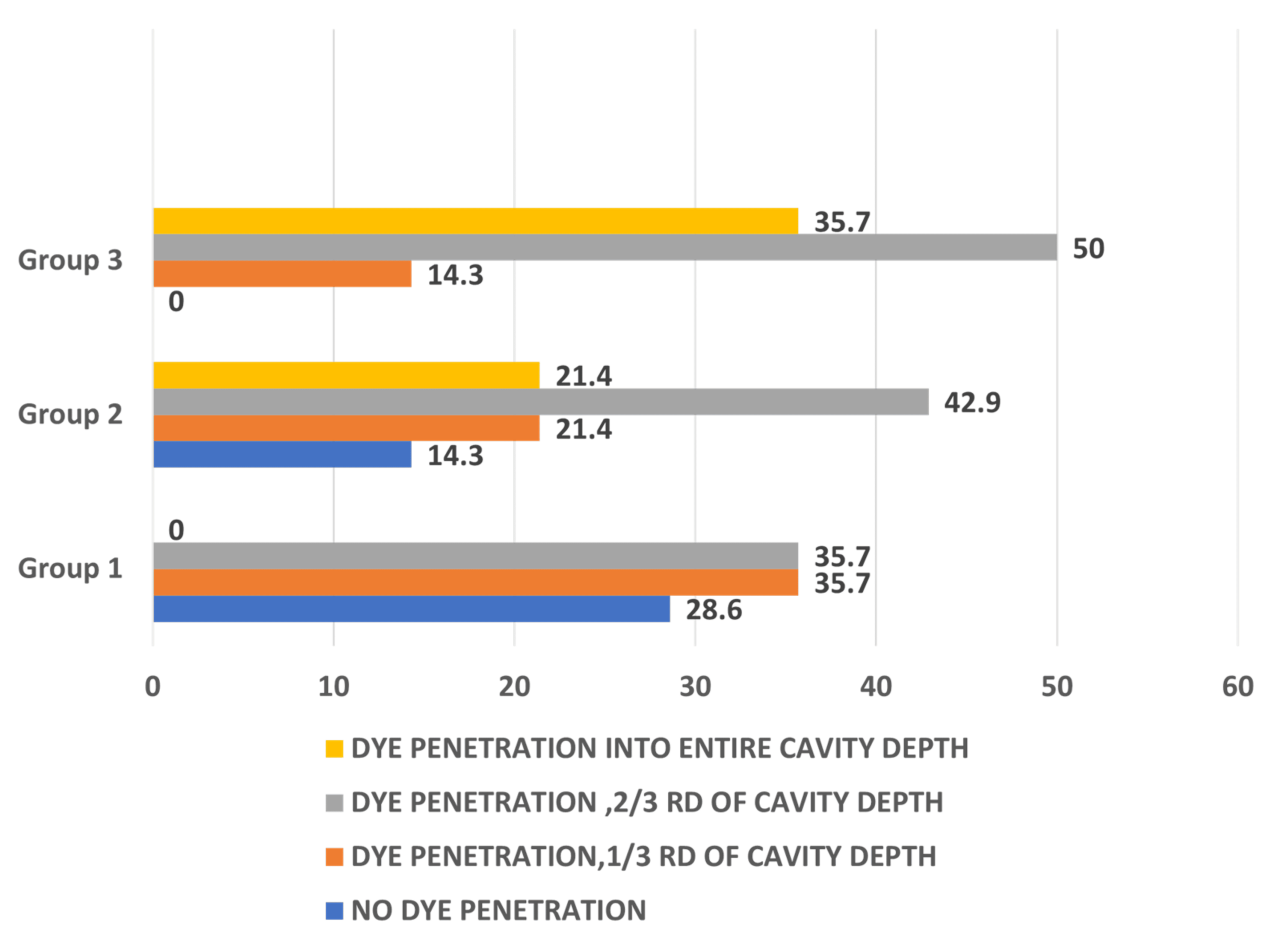
Graphical representation of comparison of microleakage scores. Group I, NeoSpectra; Group II, Filtek Z250; Group III, Tetric EvoCeram bulk fill. Score 0, no dye penetration; score 1, dye penetration less than one-third of the cavity depth; score 2, dye penetration less than two-thirds of the cavity depth; score 3, dye penetration into the entire cavity depth.

Figure 3 shows that score 3 expression was highest in Group III with Tetric EvoCeram, while NeoSpectra primarily exhibited scores 1 and 2.

## Discussion

Resin composites have been extensively used in the field of restorative dentistry due to their aesthetic features and bonding ability to tooth structures. Annual failure rates of composite resin restorations range from 3% to 11%, according to recent literature [10]. According to a 2015 meta-analysis by Beck et al. on the 10-year survival rate of composite restorations, approximately 5% of failures were attributed to bulk fracture and 8% to significant wear [11].

The majority of the failure rate is mainly attributed to the secondary caries and marginal failure of the restoration [10]. The restoration-tooth margin is the most prone site to such recurrent caries. Emphasis on the solidity of the restoration at the resin-tooth interface plays a salient role in the longevity of the restoration. However, this interface is a physically vulnerable area, liable to microleakage and secondary caries formation. These interfacial defects at the margin may develop momentarily due to the stresses developed in the restoration due to polymerization shrinkage or as a result of long-term exposure to thermal and mechanical stresses [12].

Dental resin composite material is predominantly made up of an organic resin matrix that comprises multi-reactive monomers and photoinitiators, reinforced by inorganic or organic fillers. The fillers, constituting the dispersed phase of composites, serve to occupy the interstices between the resin matrix, thereby providing mechanical reinforcement [5]. Various parameters pertaining to fillers, including quantity, physical size, configuration, and distribution, exert a significant influence on the physical and mechanical properties of composites. Besides the enhancement of the strength, the simultaneous mitigation of shrinkage that occurs during polymerization is also an added advantage of the filler particles. Moreover, these fillers aid in reducing thermal stress-induced expansion, water sorption, and staining properties, thereby improving the overall workability of the composite [6].

This study emphasizes the importance of fillers and nanotechnology that, together, overcome the disadvantages of macrofilled and microfilled resin composites. It is a comparison of the marginal adaptation and micropermeability of two nanohybrids and a pre-polymerized resin filler group.

The evaluation of marginal adaptation was performed using a scanning electron microscope, according to Blunck and Zaslansky's criteria, which are based on the gap evident between the tooth surface and the restoration. SEM has been chosen for its superior imaging resolution and capacity to provide detailed insight into the interface between the tooth and the restoration. The microleakage was assessed using a stereomicroscope, which provides a clear two-dimensional view of the resin-dentin interface to evaluate the depth of dye penetration. Moreover, it is a simple and effective method that has enhanced visibility from illumination options.

A significant proportion of Class II cavities present with gingival margins located apical to the CEJ, involving dentine and/or cementum. Consequently, the cervical margins of restorations are often placed on these substrates, which may compromise the marginal seal compared to placements on enamel [13]. Hence, in this study, the gingival margin was placed 1 mm below the CEJ.

According to a study, cervical margins showed more evidence of microleakage than the occlusal margins with and without liner because of the difficulty in the adaptation of the stiffer materials at the cavosurface margin [10,13]. Hence, the leakage in this study was assessed relative to the cavosurface margin.

A study regarding the marginal adaptation of Class II restorations using bulk-fill resin composites of different viscosities (condensable and flowable) using micro-CT showed that there was no statistical difference in most of the groups between paste-like and flowable resins. The flowable composites outperformed their condensable counterparts and produced an enhanced marginal adaptation. They concluded that the composition of the material plays an important role in marginal adaptation rather than the viscosity of the material and that bulk-fill composites could be potential alternatives to conventional composite resin in improving the marginal adaptation [14].

Numerous studies in the literature have demonstrated a correlation between filler parameters and the mechanical properties, aesthetics, and handling characteristics of composites [15-17]. There has been a tremendous improvement in filler technology in the past two decades, especially a consistent reduction in the size of filler particles, and variations in their incorporation have been utilized in different brands of composite materials. The evolution from hybrid and micro-hybrid composites to microfilled, and more recently to nanosized composites, has been well documented. Currently, the market is dominated by two categories of nanosized composites: nanofilled and nanohybrid composites [7].

The concept of decreasing the size of filler particles resulted in an increased surface area, thus leading to heightened interfacial surface energy between the filler and matrix components, which contributes to the improvement of flexural strength. Dental composites integrating nanoparticles exhibit superior mechanical properties, including flexural strength, compressive strength, and wear resistance, when compared to conventional micro-composites. Additionally, higher filler content correlates with reduced polymerization shrinkage of the resin, thereby improving cavity wall adaptation and minimizing the area susceptible to biofilm penetration at the interface. They can also withstand translational/rotational movements, which enhances the composure of the matrix phase [18-20].

The results of the study can be correlated with various studies in the literature, which have proved the role of increased filler quantity in the reduction of polymerization shrinkage. This could be due to less availability of the monomer in the resin for shrinkage [21,22].

The composite used in this study, NeoSpectra ST, based on pre-polymerized or partially polymerized resin fillers (PPRF), reduces polymerization shrinkage and improves polishability compared to conventional resin composites containing traditional fillers [23,24].

In scanning electronic microscopic evaluation of the marginal adaptation, the Tetric EvoCeram produced inferior results with a greater number of samples exhibiting MQ4 scoring, which indicates a severe marginal gap, greater than 2 µm, with severe marginal irregularities. The NeoSpectra ST produced better results, with maximum samples having MQ1 and MQ2 scoring with no gap at the interfaces in either case, with or without marginal irregularity.

Despite the positive results obtained from the present study, these PPRFs exhibit a deficiency in active binding sites for surface coupling, thereby diminishing their ability to form strong bonds with the resin matrix. This deficiency may result in the separation of the matrix from the fillers, ultimately compromising the mechanical properties of the restoration in the long term [25].

The samples were subjected to thermocycling of 1,000 cycles, mimicking six months of the aging process, before evaluation of microleakage. On microscopic examination, significant differences in the leakage values were not evident between the groups. However, the NeoSpectra group slightly outperformed the other two groups. This might be attributed to the advanced filler technology in this particular group, which uses pre-polymerized resin fillers. Instead of being a mere nanohybrid, it combines the benefits of a nanohybrid and the process of pre-polymerization. The low elastic modulus of the pre-polymerized fillers in the resin adds to its lower polymerization shrinkage potential. But this is in conflict with the study results, where they claim that the high filler load in the bulk fill composite resin decreases the polymerization shrinkage but increases the elastic modulus as well [26].

Limitations

This research solely employed vertical sectioning of the samples in the mesial-distal orientation for examination of marginal adaptation and leakage. However, a more precise method for assessing overall leakage across different sections is warranted. Performing highly standard assessments using micro-CT will be the future direction of this research. The effect of mechanical loading, along with thermocycling to emulate intraoral conditions, was not evaluated. The role of low modulus pre-polymerized fillers and their influence on strength should be evaluated in future studies. In vivo studies are needed to obtain a better understanding of clinical situations.

## Conclusions

Within the limitations of the study, it is concluded that NeoSpectra outperformed other composites in marginal adaptation due to its pre-polymerized filler content. In terms of microleakage, there is not much difference between the groups, which may be attributed to their lack of active bond sites. Further studies are needed to overcome the disadvantages.

## References

[1] Pizzolotto L, Moraes RR (2022). Resin composites in posterior teeth: clinical performance and direct restorative techniques. Dent J (Basel).

[2] Demarco FF, Cenci MS, Montagner AF, de Lima VP, Correa MB, Moraes RR, Opdam NJ (2023). Longevity of composite restorations is definitely not only about materials. Dent Mater.

[3] Ferracane JL, Hilton TJ (2016). Polymerization stress--is it clinically meaningful?. Dent Mater.

[4] Malhotra N, Kundabala M, Shashirashmi A (2010). Strategies to overcome polymerization shrinkage--materials and techniques. A review. Dent Update.

[5] Liu J, Zhang H, Sun H, Liu Y, Liu W, Su B, Li S (2021). The development of filler morphology in dental resin composites: a review. Materials (Basel).

[6] Wang Z, Chiang MY (2016). System compliance dictates the effect of composite filler content on polymerization shrinkage stress. Dent Mater.

[7] Afolabi OA, Ndou N (2024). Synergy of hybrid fillers for emerging composite and nanocomposite materials: a review. Polymers (Basel).

[8] Blunck U, Zaslansky P (2007). Effectiveness of all-in-one adhesive systems tested by thermocycling following short and long-term water storage. J Adhes Dent.

[9] Hui LE, Thomas MS, Jathanna V (2019). Effect of caries-detecting dye on microleakage of composite resin restorations bonded with total-etch and self-etch adhesive systems. J Clin of Diagn Res.

[10] Opdam NJ, van de Sande FH, Bronkhorst E (2014). Longevity of posterior composite restorations: a systematic review and meta-analysis. J Dent Res.

[11] Beck F, Lettner S, Graf A (2015). Survival of direct resin restorations in posterior teeth within a 19-year period (1996-2015): a meta-analysis of prospective studies. Dent Mater.

[12] Sun J, Eidelman N, Lin-Gibson S (2009). 3D mapping of polymerization shrinkage using X-ray micro-computed tomography to predict microleakage. Dent Mater.

[13] Bogra P, Gupta S, Kumar S (2012). Comparative evaluation of microleakage in class II cavities restored with Ceram X and Filtek P-90: an in vitro study. Contemp Clin Dent.

[14] Baltacioğlu İH, Demirel G, Öztürk B, Aydin F, Orhan K (2024). Marginal adaptation of bulk-fill resin composites with different viscosities in class II restorations: a micro-CT evaluation. BMC Oral Health.

[15] Özdemir S, Ayaz İ, Çetin Tuncer N, Barutçugil Ç, Dündar A (2025). Evaluation of polymerization shrinkage, microhardness, and depth of cure of different types of bulk-fill composites. J Esthet Restor Dent.

[16] Islam MS, Nassar M, Elsayed MA, Jameel DB, Ahmad TT, Rahman MM (2023). In vitro optical and physical stability of resin composite materials with different filler characteristics. Polymers (Basel).

[17] Oliveira GU, Mondelli RF, Charantola Rodrigues M, Franco EB, Ishikiriama SK, Wang L (2012). Impact of filler size and distribution on roughness and wear of composite resin after simulated toothbrushing. J Appl Oral Sci.

[18] Maran BM, de Geus JL, Gutiérrez MF (2020). Nanofilled/nanohybrid and hybrid resin-based composite in patients with direct restorations in posterior teeth: a systematic review and meta-analysis. J Dent.

[19] Randolph LD, Palin WM, Leloup G, Leprince JG (2016). Filler characteristics of modern dental resin composites and their influence on physico-mechanical properties. Dent Mater.

[20] Takahashi H, Finger WJ, Wegner K, Utterodt A, Komatsu M, Wöstmann B, Balkenhol M (2010). Factors influencing marginal cavity adaptation of nanofiller containing resin composite restorations. Dent Mater.

[21] Lopez C, Nizami B, Robles A, Gummadi S, Lawson NC (2024). Correlation between dental composite filler percentage and strength, modulus, shrinkage stress, translucency, depth of cure, and radiopacity. Materials (Basel).

[22] Kaur G, Bansal RK, Bansal M, Bansal D, Garg R, Singla S, Gupta S (2024). Evaluation of marginal adaptation of SDR Plus, fiber-reinforced, and nanofilled composites in endodontically treated teeth: a scanning electron microscopic study. Cureus.

[23] Salazar DC, Dennison J, Yaman P (2013). Inorganic and prepolymerized filler analysis of four resin composites. Oper Dent.

[24] Blackham JT, Vandewalle KS, Lien W (2009). Properties of hybrid resin composite systems containing prepolymerized filler particles. Oper Dent.

[25] Gurgan S, Koc Vural U, Miletic I (2022). Comparison of mechanical and optical properties of a newly marketed universal composite resin with contemporary universal composite resins: an in vitro study. Microsc Res Tech.

[26] Rizzante FA, Mondelli RF, Furuse AY, Borges AF, Mendonça G, Ishikiriama SK (2019). Shrinkage stress and elastic modulus assessment of bulk-fill composites. J Appl Oral Sci.

